# A cohort study of sustainable cultivation methods in mandarin orange orchards across Japan

**DOI:** 10.5511/plantbiotechnology.25.0605a

**Published:** 2025-12-25

**Authors:** Fuki Fujiwara, Yukari Okano, Daisuke Takata, Hayato Maruyama, Ryota Arakawa, Natsuko I Kobayashi, Kie Kumaishi, Megumi Narukawa, Yui Nose, Tsuyoshi Isawa, Takuro Shinano, Kae Miyazawa, Naoto Nihei, Yasunori Ichihashi

**Affiliations:** 1 RIKEN BioResource Research Center, Koyadai, Tsukuba, Ibaraki 305-0074, Japan; 2 RIKEN Center for Sustainable Resource Science, Koyadai, Tsukuba, Ibaraki 305-0074, Japan; 3 Graduate School of Agricultural and Life Sciences, The University of Tokyo, Yayoi, Bunkyo-ku, Tokyo 113-8657, Japan; 4 Faculty of Food and Agricultural Sciences, Fukushima University, Kanayagawa, Fukushima, Fukushima 960-1296, Japan; 5 Research Faculty of Agriculture, Fundamental AgriScience Research, Hokkaido University, Kita-9 Nishi-9, Kita-ku, Sapporo, Hokkaido 060-0808, Japan; 6 Research Institute of Environment, Agriculture, and Fisheries, Osaka Prefecture, Habikino, Osaka 583-0862, Japan; 7 Mayekawa Research Institute Co., Ltd., Koto-Ku, Tokyo 135-8482, Japan; 8 The Fukushima Institute for Research, Education and Innovation (F-REI), Yazawa, Gongendo, Namie, Futaba, Fukushima 979-1521, Japan

**Keywords:** cohort study, mandarin orange, propensity score, real-world data, sustainable agriculture

## Abstract

Variability in environmental conditions and farming practices often leads to discrepancies between experimental results and outcomes in farmers’ fields. This gap poses a challenge for understanding the effects of agricultural inputs and methods under real-world conditions, particularly in fruit cultivation systems, where large-scale experimental data are limited. In this study, we applied a cohort study approach leveraging data from farmers’ fields to investigate the effects of pesticide and fertilizer application methods on fruit quality and soil properties in mandarin orange orchards. Biases arising from differences in covariates among cultivation methods were controlled using the inverse probability weighting (IPW) based on propensity scores. Consequently, compared to local-scale analysis between adjacent fields, the nationwide cohort analysis detected a greater number of significant effects of cultivation methods by utilizing its larger sample size. Through this analysis, we found important insights into the effects of pesticide and fertilizer application methods on plant pathogens, nutritional quality, and soil properties in sustainable cultivation systems of mandarin orange. This study demonstrates that cohort analyses using real-world data have great potential to advance agricultural biotechnology by providing effective feedback from farmers’ fields and bridging the gap between scientific research and real-world agriculture.

## Introduction

Today’s agriculture is underpinned by advancements in biotechnology, including the development of cultivars, fertilizers, pesticides, and biostimulants. One of the challenges of applying them is the uncertainty of their effects in actual farmers’ fields due to the variability in field conditions and farmers’ practices. For example, the performance of agricultural chemicals can differ depending on environmental factors, such as temperature and soil properties (Chaplain et al. 2011; Maliszewska and Tęgowska 2017), and practical factors, such as sprayer characteristics (Ebert and Downer 2006). These differences often lead to a gap between results from controlled experiments and outcomes in actual fields (Davidson et al. 1967; Nkebiwe et al. 2024). Thus, considering the variability of real-world conditions is important for understanding and improving the effectiveness of agricultural inputs and methods in farmers’ fields.

**Figure 1:**
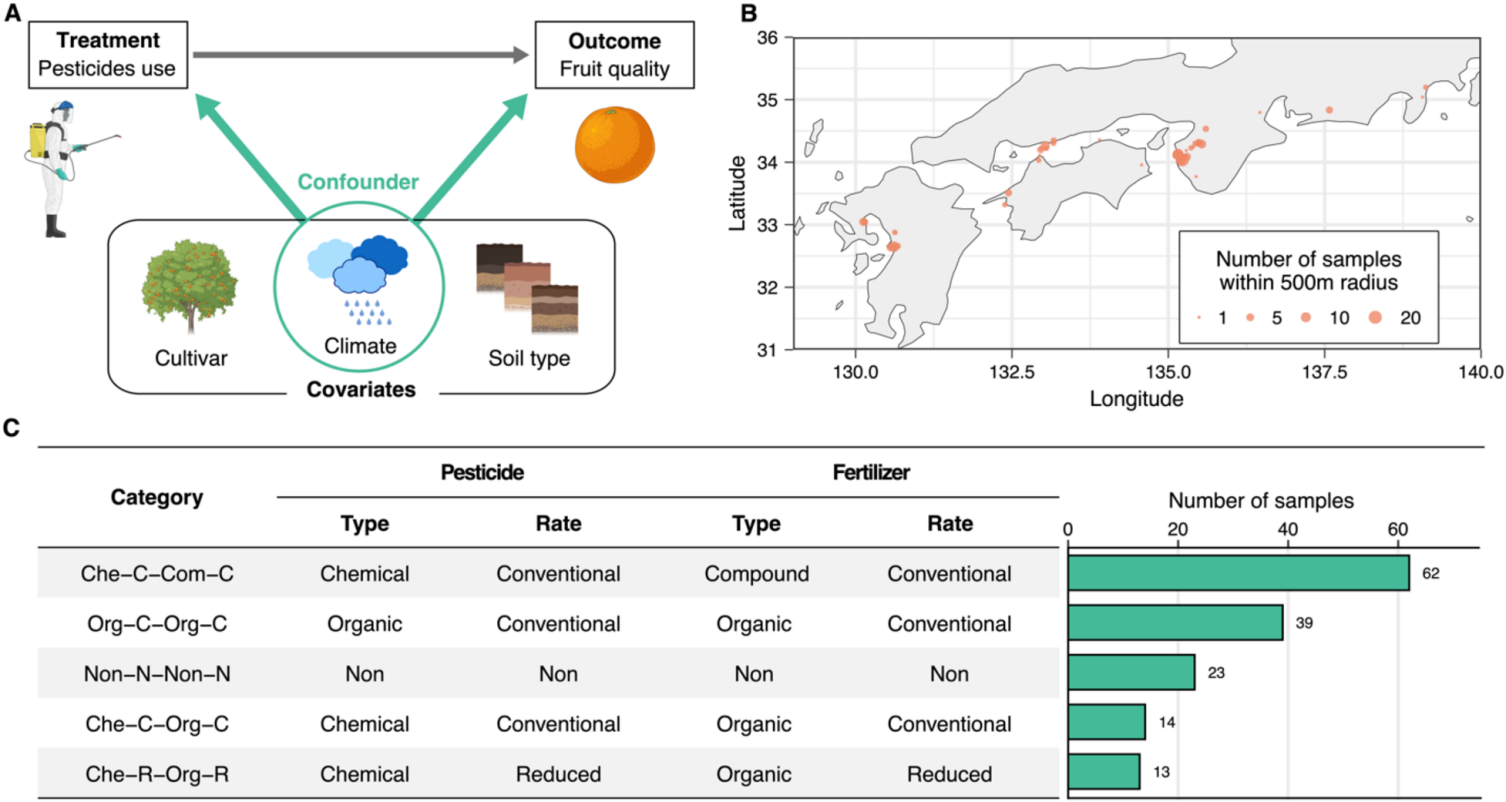
Figure 1. Concept, sampling sites, and cultivation methods of this cohort study. (A) Example of covariates and confounders. In this example, climate is a confounder affecting both the treatment assignment and outcome. (B) Locations of sampling. Sampling sites within a 500 m radius are represented by a single point. Point size shows the number of samples in each radius. (C) Definition and sample size of each cultivation method categorized based on pesticide and fertilizer application. The top five categories are shown. All categories are listed in Supplementary Table S1.

One promising approach to account for real-world variability is observational studies leveraging data from farmers’ fields. These data can capture various environmental conditions and practices without the high cost of experiments. This is particularly useful for studies of fruit cultivation systems, which require long cultivation periods. However, observational studies face challenges in evaluating the effects of particular inputs or methods, because these “treatments” are not randomly assigned but chosen by farmers. This introduces bias arising from confounders, which affect both treatment selection and outcomes (Figure 1A). Therefore, addressing confounding bias is crucial to fully utilize data from farmers’ fields.

Such confounding biases can be addressed using appropriate statistical methods, even when the confounders are not directly identifiable. These methods adjust covariates, which are considered potential confounders (Figure 1A). One simple method is to select a subset of samples with identical values for observed covariates (Scarnecchia 1988). However, due to the difficulty in finding such samples, this method often results in a limited sample size. To address this limitation, a more efficient method has been developed, which summarizes multiple covariates into a single value called a propensity score and aligns the distributions of this score across groups to be compared (Rosenbaum and Rubin 1983). While this method has been applied in epidemiology and economics, where experimental studies are often impractical due to cost or ethical concerns, it has rarely been adopted in plant science and agronomy. Therefore, this method has the potential to provide valuable feedback on the effectiveness of biotechnologies in real-world conditions and offer meaningful insights for their improvement.

To demonstrate the utility of real-world data analysis in biotechnology research, we conducted a cohort study: an observational study designed to follows sample groups receiving different treatments. We focused on mandarin orange cultivation, which requires a long cultivation cycle and poses practical challenges for large-scale experiments. Because sustainable cultivation practices are a key issue in mandarin orange production, this study investigates the effects of various pesticide and fertilizer application methods on fruit quality and soil properties using real-world data. By integrating field data with statistical methods for cohort studies, we explore the potential of real-world data as a valuable source of information for evaluating the effectiveness of applied technologies in agricultural fields.

## Materials and methods

### Collection of cohort samples

We collected fruit and soil samples from mandarin orange orchards across 12 prefectures of Japan (Figure 1B). These samples were collected during the harvest season between November and February in 2021 and 2022. The soil samples were taken from four spots located directly beneath the outer perimeter of the tree canopy. The top 5 cm of soil was removed, and the next 10 cm of soil was sampled for soil analysis. At least 10 fruit samples were collected from the same trees used for soil sampling. Information about orchards, including pesticides and fertilizer use, cultivar, and tree age, was also recorded. Cultivars were classified into four groups: extra-early, early-, mid-, and late-maturing cultivars. By using the coordinates, soil type was identified based on the Comprehensive Soil Classification System of Japan obtained from the dataset provided by Japan Soil Inventory (Obara et al. 2015). Annual mean temperature, total precipitation, and mean sunshine duration were obtained from the Agro-Meteorological Grid Square Data (Ohno et al. 2016). For detailed information about each sample, see Supplementary Dataset1.

### Paired-field study

For comparison with the cohort samples, we identified one pair of adjacent fields with different cultivation methods at Kainan-city, Wakayama, Japan (34°8′N, 135°9′E). One field received chemical pesticides and chemical-organic compound fertilizers both at conventional rates, while the other field received chemical pesticides and organic fertilizers at reduced rates. The soil and fruit samples were collected at the harvest season in 2021 and 2022.

### Fruit quality analysis

The severity of citrus melanose was evaluated based on the standard method in Japan (MAFF 2016). Each sample was assigned a grade: “A” (score=5) for fruits with clearly visible lesions, “B” (score=1) for fruits with sporadically visible lesions, and “C” (score=0) for fruits with no lesions on the surface. The disease severity was calculated from 4–19 fruits per orchard using the following formula: Σ (number of samples in each severity grade × score)×100÷(total number of surveyed samples × 5).

The color of fruit peel was measured using a CIELAB color reader (CR-13, Konica Minolta Sensing Inc., Osaka, Japan) that measured the L*, a*, and b* values of CIE (Commission International de l’Eclairge) 1976 L*a*b* color space units (CIELAB system). Hardness was determined at three longitudinal points using a hand-held penetrometer (KM-5, Fujiwara Scientific Co., Ltd., Tokyo, Japan) equipped with a core-shaped probe. Soluble solids content (Brix) and titratable acidity (Acid) were measured using a refractometer (PAL-BX/ACID1, ATAGO Co., Ltd., Tokyo, Japan) after squeezing juice from half the pulp of each fruit.

The nitrogen concentration in mandarin orange juice was measured using the semi-micro Kjeldahl method. First, 40 mg of ground freeze-dried samples was digested with sulfuric acid and hydrogen peroxide. Then, ammonia was trapped using a Kjeldahl distillation unit (Buchi 323 Distillation Unit) and quantified by titration.

### Soil chemical properties analysis

Soil samples were air-dried in a greenhouse for one week and sieved through a 2 mm mesh before analysis of chemical properties. Soil pH and electrical conductivity (EC) were measured using a 1 : 5 (w/v) ratio of soil to deionized water. Carbon and nitrogen contents were measured using an elemental analyzer (vario MAX cube, Elementar Analysensysteme, GmbH, Germany). For determination of ammonium nitrogen (NH_4_-N) and nitrate nitrogen (NO_3_-N), soil samples were extracted with 2 M KCl and these concentrations were determined with a flow injection analyzer (Auto Analyzer QuAAtro 39, BL TEC K.K., Tokyo, Japan). Available phosphorus (P) was measured by the Truog method (Truog 1930). The cation-exchange capacity (CEC) was determined using 1 M ammonium acetate solution at pH 7.00, and the extractants were filtered, diluted appropriately, and measured with ICP-MS (NexION 350S, PerkinElmer, Waltham, USA) to determine exchangeable mineral elements: aluminum (Al), boron (B), barium (Ba), calcium (Ca), cadmium (Cd), cobalt (Co), cesium (Cs), copper (Cu), iron (Fe), potassium (K), magnesium (Mg), sodium (Na), manganese (Mn), molybdenum (Mo), nickel (Ni), rubidium (Rb), strontium (Sr), zinc (Zn).

### Soil microbiome analysis

DNA was extracted from freeze-dried soil samples. The library for sequencing was prepared through a two-step PCR amplification, targeting the V4 region of the bacterial 16S rRNA gene and the Internal Transcribed Spacer (ITS) regions of the fungal rRNA gene. Sequencing was conducted using Illumina MiSeq. Subsequent bioinformatic analysis was performed using Qiime2 software. For detailed information on these procedures, see Supplementary Methods.

### Cultivation method categories based on pesticide and fertilizer application

Cultivation practices were categorized based on the type and application rate of pesticides and fertilizers (Figure 1C, Supplementary Table S1). Each method was assigned a code consisting of four components: (1) pesticide type (Che: chemical, Org: organic, Non: no pesticide), (2) pesticide application rate (C: conventional, R: reduced, N: none), fertilizer type (Che: chemical, Com: chemical-organic compound, Org: organic, Non: no fertilizer), and (4) fertilizer application rate (C: conventional, R: reduced, N: none). Organic pesticides are the pesticides permitted in organic farming in Japan, such as Bordeaux mixture and elemental sulfur. Application rates of pesticides and fertilizers were determined based on the standard pesticide use frequency and standard nitrogen application rates, respectively, for mandarin orange cultivation in each orchard’s region.

### Data analysis for cohort data

**Figure 2:**
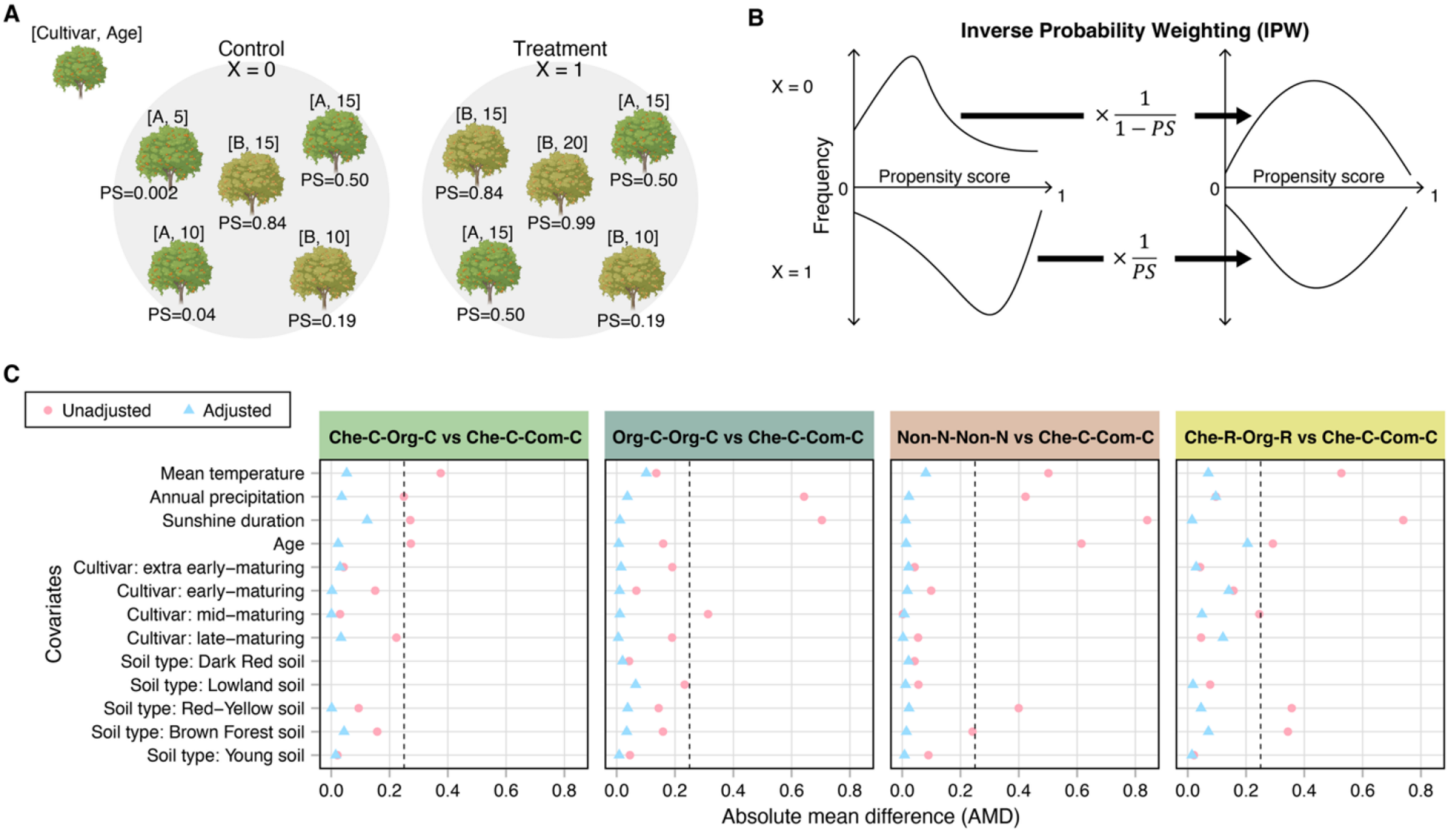
Figure 2. Covariate adjustment using IPW. (A) Example of propensity scores in a comparison between two groups. The PS is calculated as a probability of receiving a treatment (X=1) given tree cultivar and age. (B) Creation of pseudo-population using IPW to balance the distribution of covariates. (C) AMD for each covariate between each cultivation method and the conventional method before and after covariate adjustment. Dashed line indicates AMD=0.25.

The effects of each pesticide and fertilizer-based method were estimated after adjusting the covariates using inverse probability weighting (IPW). The covariates included cultivar category, tree age, annual mean temperature, precipitation, sunshine duration, and soil type. The target variables included fruit quality, soil chemical properties, soil ions, microbial diversity, abundance of bacterial and fungal genera, and abundance of bacterial functional groups. In IPW, firstly, we calculated the propensity score (PS) for each sample, which is the probability of receiving a treatment given a set of covariates (Figure 2A). Then, the weight for each sample was calculated as the inverse of the PS to generate a pseudo-population in which the covariates are equally distributed between the compared groups (Figure 2B). For evaluation of this adjustment, c-statistics of the PS and absolute mean difference (AMD) of the covariates were calculated. The standardized effect sizes of each cultivation method on the target variables were estimated as the standardized regression coefficient (β) in weighted linear regression models using the weight calculated in IPW. For more detailed information on these analyses, see Supplementary Methods.

### Data analysis for paired-field data

In paired-field data, the effects of the treatment (Che-R-Org-R) were estimated based on the standardized regression coefficient from linear mixed models to consider the repeated measures for the same tree in 2021 and 2022. In this model, the cultivation method and sampling year were regarded as fixed effects, and individual trees were considered as random effects.

## Results

### Sample collection and covariate adjustment

We identified various cultivation methods for mandarin orange orchards across Japan (Figure 1C, Supplementary Table S1). The most predominant method among the 206 cohort samples was categorized as Che-C-Com-C, in which chemical pesticides and chemical-organic compound fertilizers were applied at conventional application rates. Thus, this method was defined as the conventional method (control) to estimate the effects of other cultivation methods.

The c-statistic of PS exceeded 0.8 (Supplementary Table S2), indicating that the assumption underlying the PS was satisfied (see Supplementary Methods). In addition, while all AMDs of the covariates exceeded 0.25 before covariate adjustment, they were reduced to less than 0.25 after the adjustment in the samples of four cultivation methods: Che-C-Org-C, Org-C-Org-C, Non-N-Non-N, and Che-R-Org-R (Figure 2C). The remaining cultivation methods still exhibited AMDs exceeding 0.25 after the adjustment (Supplementary Figure S1), because of a small sample size and insufficient overlap of the covariates. Thus, these methods were excluded in the estimation of their effects.

### Effects of each cultivation method

**Figure 3:**
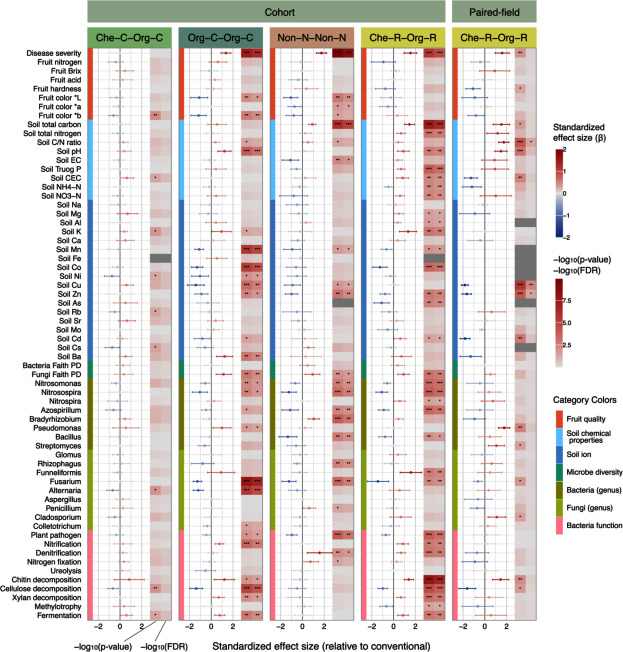
Figure 3. Effect size of each cultivation method on important variables relative to conventional method (Che-C-Com-C). Each point and error bar represents estimated standardized effect size (β) and its 95% confidence intervals for each variable. The color of points and error bars indicates the magnitude of estimated mean difference. Heatmap shows *p*-value (left column) and FDR (right) of each estimated effect. Asterisks (*) show significant effect, and gray cells indicate NA value. * <0.05, ** <0.01, *** <0.001. The results for all variables are provided in Supplementary Dataset2.

Using the weights from IPW, we estimated the standardized effect size (β) of the selected cultivation methods relative to the conventional method on the 1576 target variables. The results for all target variables are available in Supplementary Dataset2, while Figure 3 shows the results for 57 key variables including fruit quality, soil chemical properties, ionome, microbial diversity, and some microbial taxa and functional groups involved in nutrient dynamics and plant health.

Che-C-Org-C affected 302 variables with *p*-values below 0.05, among which 10 remained significant after *p*-value adjustment (FDR<0.05). Since only the type of fertilizer differed from the conventional method, the detected effects were smaller than in other methods. All variables that showed significant changes (FDR<0.05) were the relative abundance of microbial genera such as *Hyphomicrobium* (bacteria) and *Volutella* (fungi), see Supplementary Dataset2.

Org-C-Org-C affected 887 variables (*p*<0.05), with 793 retaining significance after *p*-value adjustment. Significantly increased variables included disease severity, soil pH and Ba content, fungal diversity, *Pseudomonas*, as well as bacterial functional groups involved in nitrification, chitin and xylan decomposition, and fermentation. Significantly decreased variables included fruit color scores, soil heavy metal concentrations (Mn, Co, Cu, Ni, Zn, and Cd), ammonia-oxidizing bacteria (*Nitrosomonas* and *Nitrosospira*), pathogenic fungi (*Fusarium* and *Alternaria*), and bacterial functional groups involved in plant pathogen and cellulose decomposition (FDR<0.05).

Non-N-Non-N significantly affected 819 variables (*p*<0.05), with 727 remaining significant after *p*-value adjustment. It exhibited the most pronounced increase in disease severity among all categories (β=1.75) because it used no pesticides. Other variables that showed significant increases included soil carbon content, fungal diversity, nitrogen-fixing bacteria (*Bradyrhizobium*), and a bacterial functional group associated with denitrification. In contrast, variables that significantly decreased encompassed fruit color scores, soil heavy metal concentrations (Mn, Cu, and Zn), *Nitrosomonas*, *Nitrosospira*, *Azospirillum*, *Bacillus*, and *Rhizophagus*, *Fusarium*, and a bacterial group of plant pathogens (FDR<0.05).

Finally, Che-R-Org-R significantly affected as many as 1,047 variables (*p*<0.05), and 1,001 were still significant after *p*-value adjustment. Significantly increased variables included disease severity, soil carbon and nitrogen content, pH, Truog P, NO_3_-N, minerals (Mg, Al, and K), soil Cd, fungal diversity, *Nitrospira*, *Funneliformis*, and bacterial functional groups associated with nitrification, denitrification, chitin decomposition, xylan decomposition, and fermentation. By contrast, significantly decreased variables included soil NH_4_-N, soil heavy metals (Mn, Co, Zn, and As), *Nitrosomonas*, *Nitrosospira*, *Azospirillum*, *Bacillus*, *Fusarium*, and bacterial functional groups involved in plant pathogen, cellulose decomposition, and methylotrophy (FDR<0.05).

### Comparison with paired-field analysis

**Figure 4:**
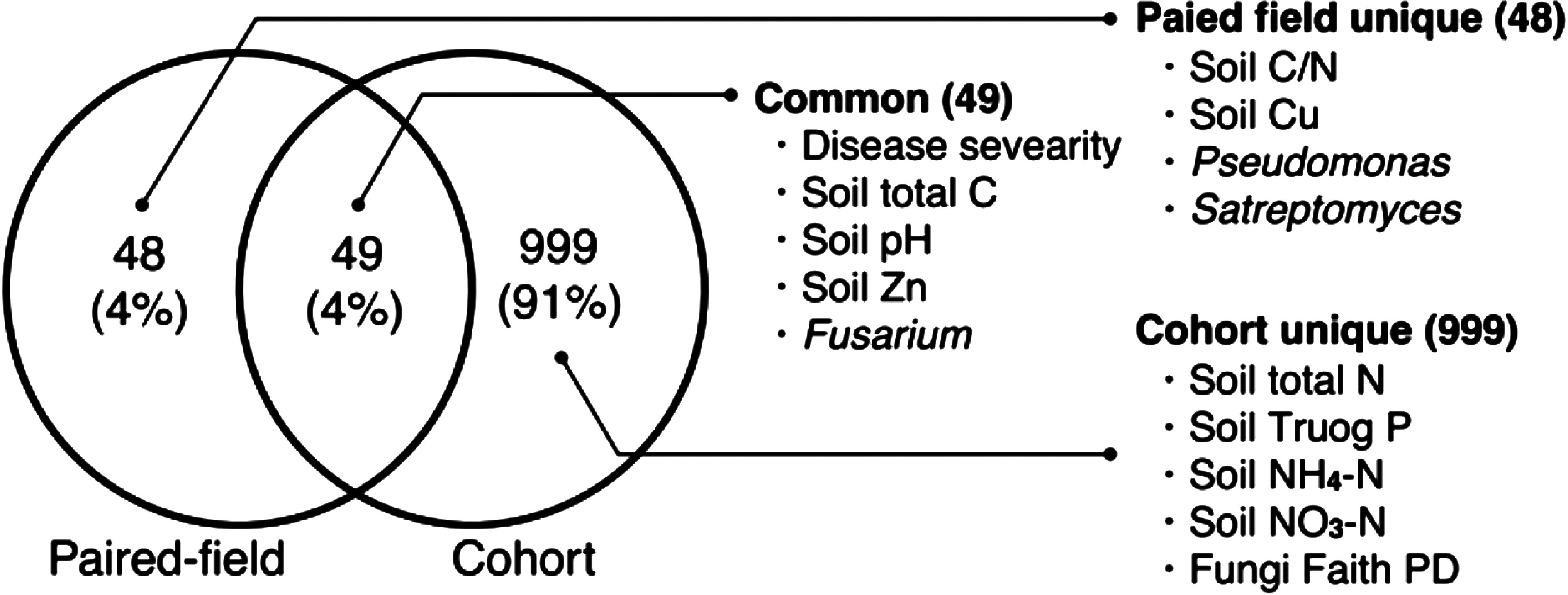
Figure 4. Overlap of significantly affected variables (*p*<0.05, with the same sign) by Che-R-Org-R in the paired-field and cohort analysis.

We finally compared the results in the cohort analysis with those in the paired-field analysis. In the paired field analysis, Che-R-Org-R affected 97 variables (*p*<0.05), with only 4 remaining significant after *p*-value adjustment (Figure 3, Supplementary Dataset3). Among the effects with *p*-value below 0.05, 49 were consistent with the effects detected in the cohort analysis (Figure 4). These included increases in disease severity, soil carbon, and pH, and decreases in Zn and *Fusarium*. Meanwhile, 48 effects were detected only in the paired-field analysis, including soil Cu, *Pseudomonas*, and *Streptomyces*. The cohort analysis detected additional effects on 999 variables, including soil nitrogen, Truog P, NO_3_-N, NH_4_-N, and microbial diversity.

## Discussion

To demonstrate the utility of real-world data in studies of plant biotechnology, we employed a cohort study approach to investigate different cultivation methods, which were categorized based on pesticide and fertilizer application methods. The orchards with different cultivation methods tended to have different covariates, especially in temperature, precipitation, sunshine duration, and tree age (Figure 2C). Since climate factors strongly affect pest infection and nutrient dynamics and thus influence farmers’ choices for cultivation practices (Delcour et al. 2015; Guo and Chen 2022), these covariates likely create confounding between cultivation methods and outcomes. Our cohort data analysis effectively reduced confounding by balancing these covariates (Figure 2C). Consequently, the cohort analysis detected a greater number of significant effects than the paired-field data analysis due to the greater statistical power from a larger sample size (Supplementary Table S2 and Figure 3). The effects detected in both analyses, such as increased disease severity and soil carbon, are considered robust effects that are less influenced by environmental factors. Meanwhile, the differences between the two analyses suggest that the local factors may generate or mask certain effects of cultivation methods (Figure 4). Taken together, this study demonstrated that cohort analyses can efficiently estimate the effects of cultivation methods by leveraging various samples, which may result in more generalizable results compared to local analyses.

Our samples included various types and levels of pesticide and fertilizer application methods (Figure 1C, Supplementary Table S1). In addition, our data included multi-layered (multi-omics) variables that are important to explain the complexity of agroecosystems (Fujiwara et al. 2023). This large-scale data enabled us to assess the effects of various pesticide and fertilizer application practices on multiple facets of agroecosystems, yielding several interesting insights beyond a mere comparison between conventional and organic farming.

First, our results suggested that pesticide use affects not only targeted foliar pathogens but also soil microbial communities. As expected, cultivation methods using fewer or no pesticides (Che-R-Org-R and Non-N-Non-N) and those that switched to organic pesticides (Org-C-Org-C) increased the damage from citrus melanose. Interestingly, these methods decreased the abundance of non-target soil-borne pathogens. This is probably due to the increased diversity of the soil fungal community, which likely contains more antagonistic microbes (Xiong et al. 2017). This is also consistent with a previous report that foliar fungicides decreased various non-target soil microbes (Santísima-Trinidad et al. 2018). In addition, our data suggested that fungal diversity is more sensitive to chemical pesticides than bacterial diversity, but not to organic pesticides. These results suggest that chemical pesticides may cause a potential trade-off between aboveground and underground plant health.

Second, our data showed no significant difference in nutritional quality among cultivation methods. This is inconsistent with previous reports that Brix and acidity of organically grown citrus fruits differed from the conventionally grown fruits (de Castro et al. 2014; Letaief et al. 2016, Sánchez-Bravo et al. 2022). This indicates that the effects on nutritional quality may vary depending on the environmental conditions across Japan. Notably, natural farming (Non-N-Non-N) did not decrease fruit and soil nitrogen levels despite no nitrogen inputs. This is likely because weeds are generally unmanaged in natural farming, which activates nitrogen fixing bacteria in legume weed roots, such as *Bradyrhizobium*. Therefore, our data suggest that the nutritional quality of mandarin oranges needs to be considered in light of the interactions between the cultivation methods and the environments.

Third, we identified significant effects on soil properties. Soil carbon content increased under reduced pesticide and fertilizer use (Che-R-Org-R) and natural farming (Non-N-Non-N) but remained unchanged under methods using organic fertilizer at the conventional rate (Che-C-Org-C and Org-C-Org-C). These results suggest that decreasing nitrogen input is more important for increasing soil carbon stock than merely increasing the amount of organic matter inputs. This may be because nitrogen input stimulates soil organic carbon decomposition (Khan et al. 2007). In addition, reducing chemical pesticides (Che-R-Org-R) or switching to organic pesticides (Org-C-Org-C) significantly reduced some heavy metal concentrations (Ni, Co, Cu, and Zn), likely due to increased pH, which reduces the solubility of these elements (Chuan et al. 1996). These results suggest that differences in cultivation methods cause significant changes in soil properties, which have important implications for crop growth and environmental impacts of sustainable cultivation systems.

Despite these valuable insights, our analysis has several limitations. First, we could not account for certain potential confounders, such as cultivation methods of neighboring orchards, microclimatic variations depending on orchard topology, and farmers’ detailed practices, due to the difficulty of data collection. According to the c-statistic, we found no evidence suggesting the presence of unobserved confounders, but future studies should focus on collecting more detailed sample information. Second, the effects of several cultivation methods could not be estimated because the balance of covariates remained poor even after adjustment (Supplementary Figure S1). This indicates that the covariate adjustment is not a one-size-fits-all solution, especially in cases with small sample size or limited overlap of covariates, as observed in these samples. Therefore, more diverse dataset with detailed background information will improve the reliability of future studies.

The importance of real-world data is gaining greater recognition as agriculture undergoes a digital transformation, improving researchers’ access to the farmers’ field data. However, these data differ from experimental data in terms of causal analysis, and thus applying an appropriate study approach is crucial to avoid misleading conclusions. The cohort analysis approach applied in this study is a promising approach to close the gap between the results of experiments and actual fields in studies for pesticides, fertilizers, cultivars, and biostimulants. This approach will advance plant biotechnologies by providing important feedback from the real world.

## Abbreviations

AMD: absolute mean difference
IPW: inverse probability weighting
PS: propensity score

## Accession numbers

The microbiome data are deposited in the NIH database (BioProject number: PRJNA1242422).

## Description of supplementary files

Supplementary Methods. Detailed description of soil metagenome amplicon sequencing and Inverse probability weighting based on propensity score.

Supplementary Table S1. Descriptions of all cultivation method categories observed in our cohort data.

Supplementary Table S2. Results of c-statistic of propensity score for each category.

Supplementary Figure S1. AMD in each covariate before and after the adjustment using IPW.

Supplementary Dataset1. Meta data and measured variables for each sample.

Supplementary Dataset2. All results of cohort analysis.

Supplementary Dataset3. All results of paired-field analysis.
