# Supplementary material for: A cohort study of sustainable cultivation methods in mandarin orange orchards across Japan
Source: Plant Biotechnol (Tokyo). 2025 Dec 25;42(4):459–66. doi: 10.5511/plantbiotechnology.25.0605a (PMC12781916; doi:10.5511/plantbiotechnology.25.0605a)
Supplement: Supplementary Data [file plantbiotechnology-42-4-25.0605a-s001.pdf]

Supplementary Methods

Soil metagenome amplicon sequencing.

Soil samples were freeze-dried using a freeze dryer (TOKYO RIKAKIKAI CO., LTD., Cat. # 63-1397-09). The freeze-dried samples were frozen using liquid nitrogen and immediately ground into a fine powder using a Multi-Beads Shocker at 3000 rpm for 15 seconds (Yasui Kikai Co., Osaka, Japan, Cat. #MB2200(S)). The DNA was extracted from 250-260 mg powder of the ground sample using an Extrap Soil DNA Kit Plus ver.2 (BioDynamics Laboratory Inc., Cat. # 212-006). The library for sequencing was prepared through a two-step PCR amplification, focusing on the V4 region of the bacterial 16S rRNA gene using 515f and 806rB primers and the Internal Transcribed Spacer (ITS) region of the fungal rRNA gene (using ITS1f and ITS2r primers), as detailed in prior studies (Caporaso et al., 2011; Ichihashi et al., 2020; Adams et al., 2013). Library concentrations were measured using an Infinite 200 PRO M Nano+ microplate photometer (TECAN Japan Co. Ltd.) and combined in equimolar ratios into a composite library. This library was then sequenced on an Illumina MiSeq system using the MiSeq Reagent Kit v3 (600-cycle, Illumina, CA, USA). Bioinformatic analyses of FASTQ files were performed using the Quantitative Insights Into Microbial Ecology 2 software (QIIME 2 version 2020.11, https://qiime2.org/). The truncation lengths were set as 200 for forward reads and 140 for reverse reads. Taxonomic classifications of 16S rRNA gene sequences were made using the Naive Bayes q2-feature-classifier, trained on the 515F/806R region with 99% operational taxonomic units (OTUs) from the SILVA 138 rRNA database. For ITS sequences, classification was performed using the classifier trained on the UNITE database for the ITS region. Sequences from archaea, eukaryotic mitochondria, and chloroplast contaminants were excluded from the final feature table. Following the taxonomic assignment of amplicon sequence variants (ASVs), the selected sequences were aligned using MAFFT and phylogenetic trees were constructed using FastTree. Based on the lowest sequence count, alpha diversity indexes and a weighted UniFrac distance matrix were calculated. Taxonomic table at the genus level was used as the taxonomic profile of soil microbiome. The profile of functional groups was also estimated using Functional Annotation of Prokaryotic Taxa (FAPROTAX), a database of ecological functions of bacteria (Louca et al, 2016).

Inverse probability weighting based on propensity score.

IPW was applied to estimate the effect of each cultivation method relative to the conventional method (Che-C-Com-C). The covariates included categorical variables (cultivar category and soil type) and numerical variables (tree age, mean temperature, precipitation, and sunshine duration). The categorical covariates were converted into dummy variables using the model.matrix function in the stats package (ver. 4.3.1). The target variables having zero values in more than half of samples were removed. The remaining variables were then subjected to log-transformation (or centered log-ratio transformation for microbiome data), followed by z-score scaling.

IPW was performed using the weightit function in the WeightIt package (ver. 1.3.2). The algorithm for calculating propensity scores was chosen as the Covariate Balancing Propensity Score (CBPS) algorithm, which estimates propensity scores while simultaneously optimizing covariate balance between treatment groups (Imai and Ratkovic, 2014). To check the assumption of "strongly ignorable treatment assignment," we calculated the c-statistic from the model used to estimate propensity scores. This assumption indicates that, after accounting for the observed covariates, treatment assignment is independent of outcomes, meaning there are no unmeasured confounders that systematically affect both treatment and outcome (Rosenbaum and Rubin, 1983). The receiver operating characteristic (ROC) curve was calculated using the roc function, and c-statistic (area under the curve) was calculated using the auc function in the pROC package (ver. 1.18.5). In addition, the balance of covariates before and after covariate adjustment was evaluated based on the absolute mean difference (AMD). The AMD for each covariate was calculated using the bal.tab function in the cobalt package (ver. 4.5.5) and visualized in a Love plot. Using the pairs of cultivation methods in which all covariates had AMDs below 0.25 after the covariate adjustment, we estimated the standardized effects of selected cultivation methods on the scaled target variables. The standardized effect size was estimated based on the standardized regression coefficient (β) in the weighted linear regression. The regression models were constructed using the svydesign in the survey package (ver. 4.4-2), in which the inverse of the propensity score (PS) was used to weight each sample. The regression coefficient (β), p-values, and 95% confidence intervals were estimated using the svyglm functions in the survey package. Finally, false discovery rates (FDR) were calculated from p-values based on the Benjamini-Hochberg (BH) method using the p.adjust function in the stats package. Our source code for these data analyses is available from a GitHub repository at https://github.com/FujiwaraFuki/cohort_agriculture.

Caporaso JG, Lauber CL, Walters WA, Berg-Lyons D, Lozupone CA, Turnbaugh PJ, Fierer N, Knight R (2011) Global patterns of 16S rRNA diversity at a depth of millions of sequences per sample. *PNAS* 108, 4516-4522

Ichihashi Y, Date Y, Shino A, Shimizu T, Shibata A, Kumaishi K, FunahashiF, Wakayama K, Yamazaki K, Umezawa A, at al. (2020) Multi-omics analysis on an agroecosystem reveals the significant role of organic nitrogen to increase agricultural crop yield. *PNAS* 117(25), 14552-14560

Adams RI, Miletto M, Taylor JW, Bruns TD (2013) Dispersal in microbes: fungi in indoor air are dominated by outdoor air and show dispersal limitation at short distances. *ISME J* 7(7), 1262–1273

Louca S, Parfrey LW, Doebeli M (2016) Decoupling function and taxonomy in the global ocean microbiome. *Science* 353: 1272-1277

Imai K, Ratkovic M (2014) Covariate balancing propensity score. *J R Stat Soc Ser B Stat Method* 76(1): 243-263

Rosenbaum PR, Rubin DB (1983) The central role of the propensity score in observational studies for causal effects. *Biometrika* 70(1): 41-55

Supplementary Table S1 Descriptions of all cultivation method categories observed in our cohort data.

Category	Pesticide		Fertilizer		Number of samples
	Type	Rate	Type	Rate	
Che-C-Com-C	Chemical	Conventional	Compound	Conventional	62
Org-C-Org-C	Organic	Conventional	Organic	Conventional	39
Non-N-Non-N	Non	Non	Non	Non	23
Che-C-Org-C	Chemical	Conventional	Organic	Conventional	14
Che-R-Org-R	Chemical	Reduced	Organic	Reduced	13
Che-R-Org-C	Chemical	Reduced	Organic	Conventional	11
Non-N-Org-C	Non	Non	Organic	Conventional	9
Che-R-Com-C	Chemical	Reduced	Compound	Conventional	6
Che-R-Com-R	Chemical	Reduced	Compound	Reduced	5
Non-N-Org-R	Non	Non	Organic	Reduced	4
Org-C-Org-R	Organic	Conventional	Organic	Reduced	4
Che-R-Che-R	Chemical	Reduced	Chemical	Reduced	2
Che-C-Che-C	Chemical	Conventional	Chemical	Conventional	1
Che-C-Com-R	Chemical	Conventional	Compound	Reduced	1
Che-C-Org-R	Chemical	Conventional	Organic	Reduced	1
Che-R-Che-C	Chemical	Reduced	Chemical	Conventional	1
Org-R-Org-C	Organic	Reduced	Organic	Conventional	1

Supplementary Table S2 Results of c-statistic of propensity score for each category.

	c-statistics
Org-C-Org-C	0.869
Non-N-Non-N	0.821
Che-C-Org-C	0.885
Che-R-Org-R	0.970
Che-R-Org-C	1.000
Non-N-Org-C	1.000

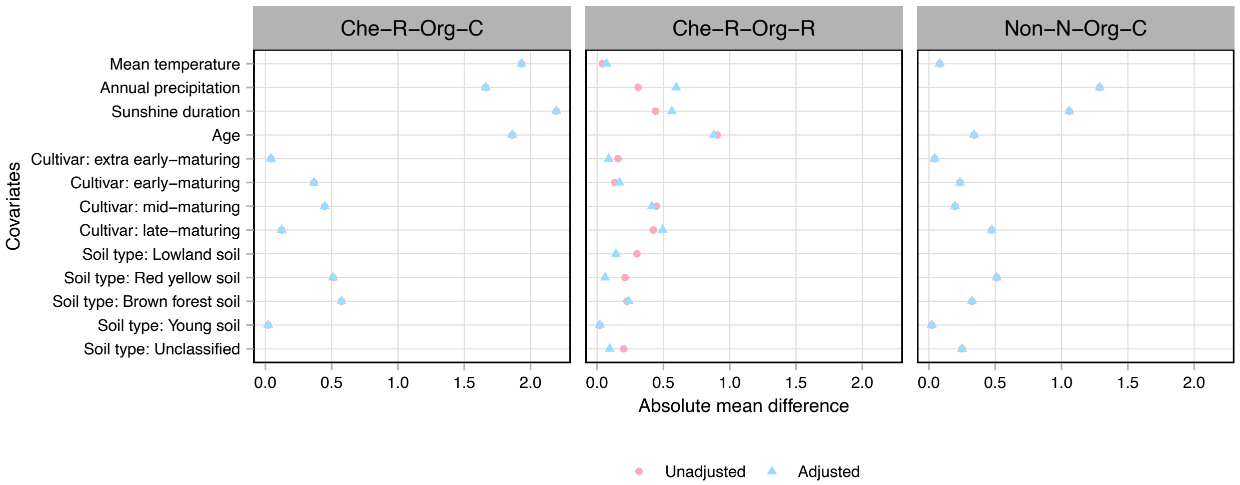

Supplementary Figure S1 AMD in each covariate before and after the adjustment using IPW.
